# Used Toothbrush as a Potential Source of Gene Expression Among Subjects With Systemic Disease and Adverse Habits

**DOI:** 10.7759/cureus.31391

**Published:** 2022-11-11

**Authors:** Manikandan G, Sujatha Govindarajan, Vishnupriya Veeraraghavan, Saranya Varadarajan, Arthi Balasubramaniam, Dharshanram R

**Affiliations:** 1 Oral Surgery, Sri Sathya Sai Medical College, Chennai, IND; 2 Oral Pathology and Microbiology, Sri Venkateswara Dental College & Hospital, Chennai, IND; 3 Research, Saveetha Dental College & Hospital, Chennai, IND; 4 Biochemistry, Saveetha Dental College & Hospital, Chennai, IND; 5 Community Dentistry, Saveetha Dental College & Hospital, Chennai, IND; 6 Community Dentistry, Dhanalakshmi Srinivasan Dental College, Chennai, IND

**Keywords:** hypertension, adverse habits, type 2 diabetes, gene expression, toothbrush

## Abstract

Purpose

To explore the viability of used toothbrush as a source of gene expression and comparison of the same among tobacco, alcohol, diabetic and hypertensive subjects.

Materials and methods

Fourteen subjects with a history of type 2 diabetes mellitus (T2DM) and hypertension were allocated to Group I, 14 subjects with tobacco and alcohol habits allocated to Group II and 14 healthy subjects allocated to Group III. Genetic materials retrieved from the used toothbrush of the study subjects were assessed for genetic expression of cyclin-dependent kinase 4 (CDK4), Bcl-2-like protein 4 (BAX), cyclin-dependent kinase inhibitor 2A (CDKN2A), B-cell lymphoma 2 (BCL2), G-protein beta-3 (GNB3), and subunit gene and transcription factor 7-like 2 (TCF7L2) by polymerase chain reaction (PCR).

Results

BAX gene showed reduced expression in tobacco and alcohol users (p=0.0497). BCL2, CDK4, and GNB3 showed no significant difference in expression in both the groups and with control. CDKN2A was expressed at a lower level in Group I and II participants. TCF7L2 showed higher expression in Group 2 participants (p=0.001).

Conclusion

The study concluded that used toothbrush is a reliable source for genetic expression. There was no difference in BCL2, CDK4, and GNB3 gene expression between subjects with systemic disease, adverse habits and healthy controls. There is a downregulation of BAX and upregulation of TCF7L2 gene in subjects with adverse habits.

## Introduction

Non-communicable diseases are chronic conditions that gradually worsen over time. They are not infective in nature. Widely recognized non-communicable diseases include cardiovascular diseases, chronic respiratory illness, cancer and diabetes. These non-communicable diseases have multifactorial causation contributed by environmental and genetic factors. The mortality rate of non-communicable diseases is high comprising 71% of death globally. Among the various diseases, mortality is high for cardiovascular diseases (17.9 million), followed by cancer (9 million) and diabetes mellitus (1.6 million) [1].

Although obesity, high cholesterol, adverse diet pattern and restrained physical activity contribute to cardiovascular diseases, hypertension accounts for the third place [2,3]. Genetics plays a major role in hypertension. Polymorphism of GNB3 gene that encodes the protein termed as guanine nucleotide-binding protein G(I)/G(S)/G(T) subunit beta-3 is a protein that has been attributed to causing cardiovascular disease [4-6]. Similarly, TCF7L2 (gene transcription factor 7-like 2) has been attributed to the pathogenesis of type 2 diabetes mellitus (T2DM) via the Wnt/b-catenin signalling pathway for regulation of adipogenesis [7]. Studies have reported that the expression of cyclin-dependent kinase inhibitor 2A (CDKN2A), has a relationship with diabetes and hypertension [8, 9].

Cancer is a challenge to human life with high morbidity and mortality. Oral squamous cell carcinoma is attributed to 90% of head and neck cancers [10]. Tobacco and alcohol are the major risk factors for oral squamous cell carcinoma that cause cancer by genetic and epigenetic alterations through complex molecular mechanisms. Early diagnosis, detection, and control is the prime strategy for improving the prognosis [11-15]. Tobacco and alcohol consumption not only causes cancer but plays a major role in the pathogenesis of diabetes and hypertension. Nicotine exposure at any age can increase insulin resistance and increase the risk of diabetes mellitus [16]. Prolonged nicotine use is associated with a greater risk of cardiovascular and autoimmune diseases [17]. Markers for cancer include apoptosis-related genes (BCL2, BAX) and mitosis-related genes (CDKN2A, CDK4). Genes such as TCF7L2 and GNB3 are linked to alcohol and nicotine addiction [18, 19].

Although saliva offers a non-invasive diagnostic specimen for genetic analysis, it has several disadvantages [20]. Recently, used toothbrush has gained importance as a reliable source of gene identification of DNA in identifying putative decedent [21]. The extraction of DNA and RNA from buccal cells of oral cavity has brought a new perspective to obtain genetic material. Compared to other methods, toothbrushes are a simpler, cost-effective method that is well tolerated by adults and children, and does not require any medical assistance. Hence, the aim of the present study was to explore the viability of used toothbrush for the differential expression of apoptosis, mitosis, and metabolic-related genes in hypertensive and diabetic patients, subjects with alcohol and tobacco habits and healthy subjects.

## Materials and methods

This cross-sectional study was implemented after obtaining ethical approval from the author’s Institution Ethical Committee (IHEC/SDC/PhD/O PATH-1759/18/398). The inclusion criteria for the study were set as patients diagnosed, under medication for diabetes and hypertension for the past five years; individuals with adverse habits of alcohol and current smokers for the past five years; healthy individuals with no adverse habits and systemic diseases. Individuals with any other systemic disease, pregnant and lactating women were excluded from the study since they may alter the genetic expression. A total of 42 volunteers who fulfilled the above-said selection criteria were allocated to the respective groups based on their diagnosis and adverse habits. All subjects were explained regarding the purpose of the study and procedures to obtain written informed consent before starting the study.

Grouping

The past medical history and adverse habit history were investigated and the subjects were grouped accordingly into three groups. The mean Ct values obtained from the pilot study with five subjects in each group were used to calculate the sample size using G power 3.1 software and one-way ANOVA test. After substituting the mean Ct values, we obtained a sample size of 42. Thus, 14 subjects with diabetes and hypertension for the past five years were allocated to Group I (DH); 14 subjects with tobacco and alcohol habits were allocated to Group II (AT) and 14 healthy subjects were allocated to Group III (Healthy).

Sample collection

Study subjects were instructed to use 0.5 g of toothpaste (Colgate) with a soft toothbrush on the day of sample collection. The toothbrushes of the subjects were gently washed and then dipped in phosphate-buffered saline. The samples were refrigerated at 4°C before being transported for processing. The bristles from the toothbrush were removed with a sterile scalpel and washed with phosphate-buffered saline [20].

RNA isolation and quantitative analysis of gene expression

Floating bristles in the collected samples were discarded and the remaining bristles of the samples were centrifuged at 3000 revolutions per minute for 10 minutes. The supernatant was discarded and the pellet was used for the isolation of RNA. Quantitative analysis of gene expression was carried out through real-time quantitative polymerase chain reaction (RT-qPCR). The RNA was extracted from the samples using a Gene Jet RNA purification kit (Thermo Scientific, Vilnius, Lithuania).

Genes assessed

Genes related to apoptosis (BCL2 and BAX), related to the cell cycle (CDK4 and CDKN2A), and metabolic-related genes such as GNB3 gene and TCF7L2 expression were assessed and compared with 18S rRNA which served as control. Table 1 demonstrates the list of genes and primers obtained from Eurofins scientific laboratory.

**Table 1:** List of Genes and their Primers

Gene	Forward Primer	Reverse Primer
BAX	5’-TCA GGA TGC GTC CAC CAA GAA G-3’	5’-TGT GTC CAC GGC GGC AAT CAT C-3’
BCL2	5’-ATC GCC CTG TGG ATG ACT GAG T-3’	5’-GCC AGG AGA AAT CAA ACA GAG GC-3’
CDK4	5’-CCA TCA GCA CAG TTC GTG AGG T-3’	5’-TCA GTT CGG GAT GTG GCA CAG A-3’
CDKN2A	5’-CTC GTG CTG ATG CTA CTG AGG A-3’	5’-GGT CGG CGC AGT TGG GCT CC-3’
GNB2	5’-GAG TCG GAC ATC AAC GCC ATC T-3’	5’-ATG CCG CAG ATG ATG CTC TCG T-3’
TCF7L	5’-GAA TCG TCC CAG AGT GAT GTC G-3’	5’-TGC ACT CAG CTA CGA CCT TTG C-3’
ACTB	5’-AGA GCT ACG AGC TGC CTG AC-3’	5’-AGC ATT TCT TCC CGG CCT TT-3’

RT-qPCR for quantitative analysis of gene expression

Multiskan SkyHigh (Thermo Scientific, Waltham, MA, USA) kit was used to quantify RNA. Isolated RNA samples (2 μg) were subjected to reverse transcription with the aid of cDNA synthesis kit (High Capacity, Applied Biosystems, Carlsbad, CA, USA). To determine the expression of each gene, 1.8 μL cDNA was used to 20 μg of the total reaction volume. Quantitative analysis of selected genes was done with 5 μL of SYBR Green PCR master mix (Applied Biosystems, Austin, TX, USA) (total reaction volume of 10 μL) on a Real-Time PCR system (Quant Studio 5, Applied Biosystems, Foster City, CA, USA). Normalization of gene expressions was done with expressions of target genes to 18S rRNA as a reference gene using the ΔΔCT method.

Data on relative ΔΔCt values of gene expression were entered into Microsoft excel (Microsoft® Corp., Redmond, WA, USA) and statistical analysis was performed using SPSS software version 23.0 (IBM Corp., Armonk, NY, USA). Comparison of gene expression among the groups was carried out using the one-way ANOVA test. Pair-wise comparison of gene expression was carried out with Tukey HSD (Honestly Significant Difference) test. The mean difference in the relative ΔΔCt values was evaluated using one-way ANOVA with Tukey’s HSD post-hoc test. A p-value ≤ 0.05 was considered to be statistically significant.

## Results

**Figure 1:**
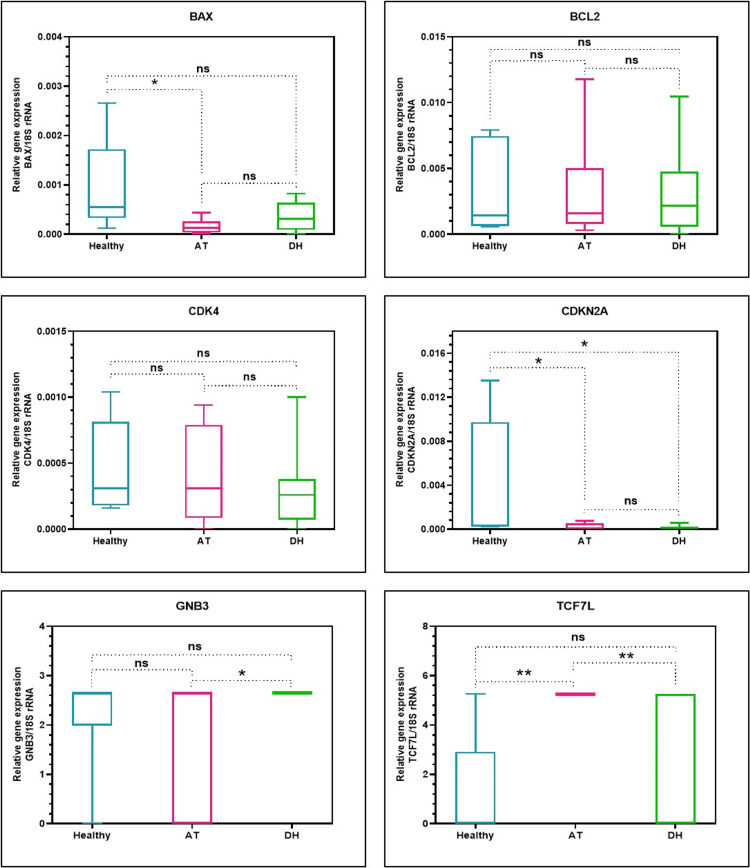
Relative gene expression analysis by RT-qPCR (Reference gene: 18S rRNA). ns: not significant, AT: Alcoholic/tobacco, DH: Diabetic/hypertension *p<0.05, **p<0.001

On comparison with the healthy control (Group III), BAX gene was downregulated in Group II (AT) (p=0.0497). Group I (DH) also showed downregulated expression with no significant difference (p=0.1398). There was downregulation of BCL2 and CDK4 in Group I (DH) and Group II (AT) in comparison with healthy controls (Group III) with no significant difference. CDKN2A gene expression was significantly downregulated in both Group I (DH) and Group II (AT) in comparison with Group III (Healthy) (p=0.0318; 0.0356). There was a significant upregulated expression of GNB2 gene in patients with diabetes and hypertension (Group I) compared to Group II (AT) (p=0.0467). However, there was no significant difference in the GNB2 gene expression between healthy controls and Group I (DH) and Group II (AT). The TCF gene expression was found to be upregulated in subjects with alcohol and tobacco habit (Group II) compared to healthy controls (Group III) and patients with diabetes and hypertension (Group I) (p=0.001; 0.0093) (Table 2 and Figure 1).

**Table 2:** Comparison of relative CT values of the genes (*statistically significant)

Marker	Healthy subjects (control)	Alcohol and Tobacco users (AT)	Diabetic and hypertensive patients (DH)	P-value (Control vs AT)	P-value (Control vs DH)	P-value (AT vs DH)
BAX	0.00093±0.00089	0.00016±0.00014	0.00034±0.00028	0.0497*	0.1398	0.1409
BCL2	0.00353±0.00325	0.00289±0.00299	0.00294±0.00284	0.708	0.7247	0.9662
CDK4	0.00046±0.00033	0.00039±0.00033	0.00029±0.00027	0.7031	0.336	0.4788
CDKN2A	0.00386±0.00525	0.00025±0.00027	0.00017±0.00018	0.0318*	0.0356*	0.4111
GNB2	2.21011±0.98838	1.63209±1.29026	2.65213±0	0.3701	0.2643	0.0467*
TCF	1.23228±1.96003	5.25359±0	2.03522±2.54482	0.001**	0.5258	0.0093*

## Discussion

The incidence of non-communicable conditions such as diabetes and hypertension has increased over the years with high morbidity and mortality rates. Also, tobacco and alcohol-related cancers of the head and neck have increased in the recent years. It is common for individuals to be addicted to both tobacco and alcohol and 65% of cancers in males are due to both tobacco and alcohol. So, we included both tobacco and alcohol users in the current study [13-15].

Early diagnosis of these conditions along with medical management and lifestyle modifications alone can decrease mortality and improve the prognosis of these conditions. Considering various diagnostic tools, the search for an accurate non-invasive tool continues. Saliva is the primary non-invasive tool for the detection of several diseases. However, it has several disadvantages such as diurnal variations and risk of contamination. Recently, toothbrushes are being explored as a source of genetic material. We aimed to compare the gene expression of apoptosis, mitosis, and metabolic-related genes in genetic material retrieved from toothbrushes used by individuals with diabetes, hypertension, tobacco, alcohol consumption and healthy controls.

We found that the expression of apoptosis-related gene (BAX gene) was significantly downregulated in subjects with alcohol, tobacco habits and patients with type 2 diabetes and hypertension. The expression of the BAX gene was significantly reduced in the AT group compared to the control group. This can be attributed to the fact that BAX gene is a pro-apoptotic gene responsible for the intrinsic pathway of apoptosis, and tobacco is known to cause apoptosis evasion leading to carcinogenesis. The BAX gene (Bcl-2 Associated X-protein) is a pro-apoptotic gene of Bcl-2 family. It plays a vital role in the intrinsic pathway of apoptosis by encoding BAX-alpha protein [22,23]. The expression of BCl-2 was slightly less in comparison with the control. However, this difference was not statistically significant. This could be attributed to the low sample size of the present study.

In the DH group, there was downregulated expression of BAX and BCL2 [24]. However, this difference was not statistically significant. This finding is consistent with an earlier report by Hasnan et al. who found upregulated BAX expression and decreased BCL2 expression in skin samples of patients diagnosed with diabetes [25]. Similar results were reported in rats with hypertension from samples taken from the left ventricle.

Cell cycle-related genes showed reduced expression in patients with alcohol and tobacco habits and patients with type 2 diabetes and hypertension. CDK4 showed no significant difference in the expression of both AT and DH in comparison with healthy controls. However, CDKN2A showed a significant reduction in its expression in both DH and AT groups compared to the control. Reduced expression of cyclins and cyclin-dependent kinases such as CDKN2A and CDK4 in specific cells have been attributed to the pathogenesis of diabetes and hypertension [8, 9]. Studies have shown that tobacco and alcohol contribute to the pathogenesis of diabetes and hypertension. Hence the decreased expression of CDKN2A in AT group may be associated with an increased risk of developing these conditions [16, 17].

Metabolic-related genes varied in expression between diabetes and hypertensive patient, and alcohol and tobacco user group. TCF expression was significantly higher in AT group. TCF showed no significant difference in expression in the DH group compared to the control. GNB2 showed no significant difference in both groups. Several studies have depicted the role of GNB2, and TCF in the pathogenesis of diabetes and hypertension and the variation of expression in tobacco and alcohol users. In our study, the relatively low sample size combined with the confounding factor of duration of tobacco and alcohol exposure could have contributed the varied results [18, 19, 26-28].

The present study demonstrated that toothbrushes are a potential source of genetic material that could be explored further for the diagnosis of diseases which is concurrent with previous studies [29-34]. However, standardization is required with a larger sample size and yield of DNA and RNA with different time intervals of toothbrushing and storage conditions. Also, accidental or purposeful exchange of toothbrushes between individuals must be avoided. The insights gained from this study may be of assistance for obtaining pure DNA samples and genomic quantification for forensic and personal identification. The limitations of the study are the small sample size and failure to assess time-dependent variations in the yield of genetic material.

## Conclusions

Our findings indicate that toothbrushes are a good source of genetic material and variations in the expression of genes among individuals with diabetes, hypertension, tobacco, and alcohol consumption were observed. Further research with a larger sample size examining a variety of genes is necessary to confirm and validate these findings. Future research can help determine accurate markers for diagnosing non-communicable diseases to reduce the incidence and prevent mortality due to these conditions.
